# Genetic Variability in Markers of HLA-C Expression in Two Diverse South African Populations

**DOI:** 10.1371/journal.pone.0067780

**Published:** 2013-07-05

**Authors:** Nikki L. Gentle, Maria Paximadis, Adrian Puren, Caroline T. Tiemessen

**Affiliations:** 1 Centre for HIV and STIs, National Institute for Communicable Diseases, National Health Laboratory Services, Johannesburg, Gauteng, South Africa; 2 Faculty of Health Sciences, University of the Witwatersrand, Johannesburg, Gauteng, South Africa; South Texas Veterans Health Care System and University Health Science Center San Antonio, United States of America

## Abstract

An insertion-deletion (indel) polymorphism within the 3′ untranslated region (UTR) of *HLA-C* has been shown to be involved in the regulation of HLA-C expression. Individuals who carry a deletion at this position exhibit increased HLA-C expression, which associates with lower viral set point in HIV-1 infected individuals. This *263* indel (rs67384697) is reported to be in strong linkage disequilibrium (LD) with a single nucleotide polymorphism (SNP) 35 kilobases upstream of *HLA-C* (*-35T/C*; rs9264942) in Caucasian individuals, making this SNP a potential marker for both HLA-C expression and HIV-1 disease progression. We therefore examined genetic variation within the *HLA-C* 3′ UTR of 265 Black and Caucasian South Africans by direct sequencing and identified haplotypes encompassing the *263* indel and another indel at position 230 in both populations. Concomitant evaluation of variability at the *−35* SNP revealed this polymorphism to be an inappropriate marker for the *263* indel in these populations. These findings provide important insights into genetic variability within the regulatory regions of *HLA-C* that have potential implications for our understanding of the regulation of HLA-C expression and its impact on HIV-1 disease progression.

## Introduction

Recently, there has been an increased interest in the role of *HLA-C* in HIV-1 infection. A single nucleotide polymorphism (SNP) 35 kb upstream of *HLA-C* has been shown to be associated with differences in HIV-1 viral set point [1] - a key early determinant of the rate of HIV-1 disease progression. This SNP (*−35T/C*; rs9264942) also strongly associates with differences in HLA-C mRNA [1], [2] and cell surface expression levels [3]. However, while these associations have consistently been shown in Caucasian cohorts [1], [3]–[7], the association between the *-35 SNP* (rs9264942) and HIV-1 viral set point has not been shown to be significant in African American cohorts - despite the presence of this SNP in this population [7]–[10]. This has lead to the suggestion that the *-35 SNP* is not the causative variant responsible for the alteration in HLA-C expression and viral set point, but rather acts as a marker in Caucasian populations for another polymorphism.

This view is supported by the findings of Kulkarni *et al*. [11], who identified a single base pair insertion-deletion (indel) polymorphism at position 263 of the *HLA-C* 3′ untranslated region (UTR) that has been shown to be in strong linkage disequilibrium (LD) with the *-35 SNP* in Caucasian individuals. They found that this variant (*263* indel; rs67384697) potentially affects the binding of a regulatory microRNA (miRNA148a) to the 3′ UTR, with a deletion at this position (*263del*) abolishing miRNA148a binding and leading to increased HLA-C expression. They also found an overrepresentation of the *263del* allele in HIV-1 controllers relative to non-controllers in a cohort of HIV-infected individuals of European ancestry, suggesting that the change in HLA-C expression associated with this allele could provide the basis for long-term protection against HIV-1 disease progression [11]. However, these data are again only representative of individuals of Caucasian ancestry and have yet to be confirmed in other populations.

That differences exist in patterns of genetic variation, and specifically in patterns of LD, between Black and Caucasian populations has become increasingly apparent. A recent study described *HLA* class I diversity in both Black and Caucasian South African populations [12], outlining the patterns of LD that characterise this region and highlighting the key differences in *HLA-C* allelic representation between these two population groups. However, no data are yet available regarding the *-35* SNP or describing variation in the *HLA-C* 3′ UTR in these populations. Here we report the first description of these data in 265 unrelated South Africans from both the Black and Caucasian population groups.

## Materials and Methods

### Study Population

A total of 265 HIV-1 negative, unrelated South African individuals were used to describe genetic variation and patterns of LD within and between the coding and regulatory regions of the *HLA-C* locus. These 168 Black and 97 Caucasian South Africans were selected from a larger previously described cohort [12] on the basis of a non-reactive HIV enzyme-linked immunosorbent assay (ELISA) test (Genscreen HIV1/2 version 2; Bio-Rad, Marnes-La-Coquette, France). Informed consent was obtained from all study participants and the study was approved by the University of the Witwatersrand Committee for Research on Human Subjects.

The DNA used for genotyping was extracted from buffy coat samples using the PEL-FREEZ DNA Isolation Kit (DYNAL Invitrogen Corporation, Carlsbad, California, USA).

### *HLA-C* Genotyping

*HLA-C* genotyping was performed at both low and high resolution as previously described [12]. However, because the genotyping was performed prior to 2005 using a single specific primer-polymerase chain reaction (SSP-PCR) genotyping method, it was possible that alleles that had not yet been identified at the time of genotyping may have been present within the sample population. Misclassification of these alleles could potentially confound LD analyses. HLA-C*02∶10 has previously been found at relatively high frequencies in other Black populations [13], however, it was not observed in the Black South African population during the prior genotyping [12]. HLA-C*02∶02 and HLA-C*02∶10 differ by only a single amino acid (T211C) in exon 4 [14], a difference that would not be detected by the SSP-PCR genotyping kit previously employed to genotype these samples. Therefore, all Black individuals who had originally been typed as having HLA-C*02∶02 alleles; as well as all individuals who were initially typed as homozygous at the *HLA-C* locus, were re-genotyped using the AlleleSEQR HLA-C PLUS Sequence-Based Typing (SBT) Kit (Abbot Molecular, Des Plaines, Illinois, USA) according to the manufacturer's instructions. Sequencing analysis and allele assignment were performed using Assign™ SBT v3.5.1 software (Conexio Genomics, Fremantle, Western Australia), with the IMGT/HLA July 2011 (v3.6.0) references. Because at least one of the alleles in any genotype combination was already known (based on the previously available genotyping data), none of the retyped samples were regarded as ambiguous.

### *HLA-C* 3′ UTR DNA Sequencing

The *HLA-C* 3′ UTR was amplified from genomic DNA using the PCR primers described by Kulkarni *et al*. [11] and the following thermocycling conditions: 94°C for 2 minutes, followed by 30 cycles of 94°C for 15 seconds, 65°C for 30 seconds, 72°C for 90 seconds and 72°C for 7 minutes. The amplicons were sequenced in both directions by capillary electrophoresis using an ABI 3100 DNA analyzer (Applied Biosystems, Foster City, California, USA) and the sequencing primers: 5′-GTGAGATTCTGGGGAGCTGA-3′ and 5′-TCTGGAAGGTTCTCAGGTC-3′. The chromatograms obtained were analysed using Sequencher v4.2 (Genes Codes Corporation, Ann Arbor, Michigan, USA) and sequences were aligned with an available reference sequence (GenBank Accession Number NG_029422) to identify polymorphic positions.

### *-35* SNP Genotyping

A real-time PCR assay was designed to genotype the *-35* SNP (rs9264942). PCR amplicons were amplified from genomic DNA using a common forward primer (5′-GCCCATACCTGTTTATACATCCA-3′) and allele-specific reverse primers (5′-CAGAAAGTCCCACAGTGCCTG-3′ and 5′-CAGAAAGTCCCACAGTGCCTA-3′). Both allele-specific primers were designed with a lock nucleic acid (LNA) modified 3′-end base. The assay was performed using the Applied Biosystems 7500 Real-Time PCR system (Applied Biosystems, Foster City, California, USA), under the following thermocycling conditions: 95°C for 10 minutes, followed by 40 cycles of 95°C for 15 seconds, 60°C for 15 seconds and 70°C for 1 minute. Reactions were performed in a 10 µl volume, containing 1x Maxima SYBR Green/ROX qPCR Master Mix (Fermentas, Burlington, Canada), 10 pmol of each primer and 20–60 ng of DNA. PCR analysis was based on calculation of ΔC_T_ (difference in cycle threshold; the difference between the C_T_ of the *-35T* and *-35C* reactions). Heterozygous individuals had ΔC_T_ values of between 0 and 0.3, while homozygous *TT* and *CC* individuals had ΔC_T_ values of ≥4 or ≤−4, respectively.

### Computational and Statistical Analyses

Allele frequencies at all polymorphic positions were determined by direct counting [15] and comparisons of these frequencies between the Black and Caucasian population groups were performed using a two-sided Fisher exact test (SISA: Simple Interactive Statistical Analysis). Deviations from Hardy-Weinberg equilibrium (HWE) were assessed using the Markov chain exact test for HWE [16]. The Excoffier-Laval-Balding (ELB) algorithm [17] was used to estimate the gametic phase of the genotypic data generated at all polymorphic positions, allowing linkage disequilibrium (LD) to be quantified as the LD coefficients [18], [19] D' and r^2^. The significance of pairwise LD between all polymorphic positions was estimated using an exact test for LD [20]. All HWE, LD and haplotypic analyses were implemented through Arlequin v3.5.1.2 [21]. All statistical measures were considered significant at p<0.05.

## Results

### HLA-C Allele Representation

Although *HLA-C* genotyping data was already available for all 265 of the individuals included in this study [12]; the development of new genotyping methods, coupled with the identification new *HLA-C* alleles in the period since the initial genotyping had been performed, made it prudent to retype approximately 20% of the samples to avoid confounding subsequent LD analyses. While all the Caucasian individuals genotyped were confirmed to be in possession of the HLA-C*02∶02 allele, the majority of the Black individuals who were initially thought to be in possession of HLA-C*02∶02 were subsequently reclassified as having HLA-C*02∶10. Additionally, an individual from the Black population group was found to be homozygous for a putative new allele (herein referred to as C*15:xx), which had previously been classified as C*15∶05. This putative new allele was found to be identical to C*15∶05:01 across exons 2, 3 and 4, with the exception of a G>A transition at nucleotide position 728 in exon 4. The alleles and their frequencies in both populations are given in Table S1.

### Genetic Variation within the *HLA-C* 3′ UTR

Direct sequencing of the 3′ UTRs of the *HLA-C* alleles present in the Black and Caucasian South African population revealed this region contained 33 polymorphisms within the approximately 400 bp of sequence analysed, including the indel at position 263 (Table S2). Thirty-one positions (including the *263* indel) were found to be polymorphic in both the Black and the Caucasian population groups (Table S2). Minor allele frequencies differed significantly between the two population groups at ten of these positions; however, these did not include the *263* indel. Positions 84 (*84G/A*; rs139211788) and 285 (*285ACTT/-*; rs60637457) were only polymorphic within the Black population. Seven other SNPs previously reported in this region were not observed in either population.

Several of the polymorphisms present in the 3′ UTR were found to only be associated with specific *HLA-C* alleles (Table 1 and 2). For the majority of alleles, these allele-specific polymorphisms were associated with the same *HLA-C* allele (or alleles) in both population groups (when the given *HLA-C* allele was present in both population groups). However, this was not always the case – as can be seen for C*02∶02 and C*02∶05. The Black population group deviated significantly from HWE at positions 46 (p = 0.026), 110 (p<0.001), 138 (p = 0.001), 267 (p = 0.023) and 303 (p<0.001), while positions 263 (p = 0.033), 266 (p = 0.032), 294 (p = 0.032), 299 (p = 0.031), 300 (p = 0.032), 307 (p = 0.024), 345 (p = 0.032) and 346 (p = 0.032) deviated significantly from HWE in the Caucasian population group.

**Table 1:** Genetic variation within *HLA-C* 3′ UTR sequences of the *HLA-C* alleles observed in the Black South African population group.

	Polymorphic Position^1^																																
	46	84	92	93	101	110	125	133	138	146	179	224	230	256	259	261	263	266	267	278	285	294	299	300	303	307	324	345	346	347	356	375	379
**Allele**																																	
**02∶02** 2	C	G	R	T	T	Y	R	K	S	Y	Y	R	I/D	M	C	T	I	C	R	R	I	A	G	T	R	C	K	A	G	S	R	T	R
**02∶05**	C	G	A	T	T	T	A	T	G	T	T	G	**I** 4	C	C	T	I	C	A	G	I	A	G	T	A	C	G	A	G	C	G	T	A
**02∶10** 2	Y^3^	R	A	T	T	Y	R	K	S	Y	Y	R	I/D	M	Y	Y	I/D	Y	R	R	I/D	M	R	W	R	Y	K	R	R	S	R	T	R
**03∶02**	C	G	A	T	T	T	G	G	C	C	C	A	D	C	C	T	I	C	G	A	I	A	G	T	G	C	T	A	G	G	A	T	G
**03∶03**	C	G	A	T	T	T	G	G	C	C	C	A	D	C	C	T	I	C	G	A	I	A	G	T	G	C	T	A	G	G	A	T	G
**03∶04** 2	C	G	A	T	T	T	G	G	S	C	C	A	D	C	C	T	I	C	G	A	I	A	G	T	R	C	T	A	G	G	A	T	G
**04∶01**	C	G	A	T	T	T	G	G	G	C	C	A	D	C	C	T	I	C	G	A	I	A	G	T	A	C	T	A	G	G	A	T	G
**04∶04**	C	G	A	T	T	T	G	G	G	C	C	A	D	C	C	T	I	C	G	A	I	A	G	T	A	C	T	A	G	G	A	T	G
**05∶01**	T	G	A	T	T	T	G	G	G	C	C	A	D	C	T	C	D	T	A	A	I	C	A	A	A	T	T	G	A	G	A	T	G
**06∶02**	C	G	A	T	T	T	G	G	G	C	C	A	D	C	T	C	D	T	A	A	I	C	A	A	A	T	T	G	A	G	A	T	G
**06∶06**	C	G	A	T	T	C	G	G	G	C	C	G	I	A	C	T	I	C	A	A	I	A	G	T	A	C	G	A	G	C	A	T	A
**06∶11**	C	G	A	T	T	T	G	G	G	C	C	A	D	C	T	C	D	T	A	A	I	C	A	A	A	T	T	G	A	G	A	T	G
**07∶01** 2	C	G	G	T	T	Y	G	G	G	C	C	G	I	A	C	T	I	C	A	A	I	A	G	T	A	C	G	A	G	C	A	T	A
**07∶02**	C	G	A	C	T	T	G	G	G	C	C	G	I	A	C	T	I	C	A	A	I	A	G	T	A	C	G	A	G	C	A	T	A
**07∶04**	C	G	A	T	T	T	G	G	G	C	C	G	I	A	C	T	I	C	A	A	I	A	G	T	A	C	G	A	G	C	A	T	A
**07∶06**	C	G	A	T	T	C	G	G	G	C	C	G	I	A	C	T	I	C	A	A	I	A	G	T	A	C	G	A	G	C	A	T	A
**07∶18**	C	G	A	T	T	C	G	G	G	C	C	G	I	A	C	T	I	C	A	A	I	A	G	T	A	C	G	A	G	C	A	T	A
**08∶01**	T	G	A	T	T	T	G	G	G	C	C	A	D	C	T	C	D	T	A	A	I	C	A	A	A	T	T	G	A	G	A	T	G
**08∶02**	T	G	A	T	T	T	G	G	G	C	C	A	D	C	T	C	D	T	A	A	I	C	A	A	A	T	T	G	A	G	A	T	G
**08∶04**	T	G	A	T	T	T	G	G	G	C	C	A	D	C	T	C	D	T	A	A	I	C	A	A	A	T	T	G	A	G	A	T	G
**12∶03**	C	G	A	T	T	T	G	G	G	C	C	A	D	C	T	C	D	T	A	A	I	C	A	A	A	T	T	G	A	G	A	T	G
**14∶02**	C	G	A	T	T	T	G	G	G	C	C	A	D	C	C	T	I	C	G	A	I	A	G	T	A	G	T	A	G	G	A	T	G
**15∶02**	C	G	A	T	T	T	G	G	G	C	C	A	D	C	T	C	D	T	A	A	I	C	A	A	A	T	T	G	A	G	A	T	G
**15∶05**	C	G	A	T	T	T	G	G	G	C	C	A	D	C	T	C	D	T	A	A	I	C	A	A	A	T	T	G	A	G	A	T	G
**15:xx**	C	G	A	T	T	T	G	G	G	C	C	A	D	C	T	C	D	T	A	A	I	C	A	A	A	T	T	G	A	G	A	T	G
**16∶01** 2	C	G	A	T	Y	T	G	G	G	C	C	A	D	C	T	C	D	T	A	A	I	C	A	A	A	T	T	G	A	G	A	C	G
**17∶01**	C	G	A	T	T	T	A	T	G	T	T	G	I	C	C	T	I	C	A	G	I	A	G	T	A	C	G	A	G	C	G	T	A
**18∶01** 2	C	G	A	T	T	T	G	G	S	C	C	A	D	C	C	T	I	C	G	A	I	A	G	T	A	C	T	A	G	G	A	T	G
**18∶02**	C	G	A	T	T	T	G	G	G	C	C	A	D	C	C	T	I	C	G	A	I	A	G	T	A	C	T	A	G	G	A	T	G

1 positions are given relative to the start of the HLA-C 3′ UTR.

2 *HLA-C* alleles C*02∶02, C*02∶10, C*03∶04, C*07∶01, C*16∶01 and C*18∶01 were found to have more than one 3′ UTR sequence.

3 where more than one allele has been observed alleles are reported using standard IUB ambiguity codes.

4 I refers to an insertion and D to a deletion.

**Table 2:** Genetic variation within *HLA-C* 3′ UTR sequences of the *HLA-C* alleles observed in the Caucasian South African population group.

	Polymorphic Position^1^																																
	46	84	92	93	101	110	125	133	138	146	179	224	230	256	259	261	263	266	267	278	285	294	299	300	303	307	324	345	346	347	356	375	379
**Allele**																																	
***01∶02***	C	G	A	T	T	T	G	G	G	C	C	A	D^4^	C	C	T	I	C	G	A	I	A	G	T	A	C	T	A	G	G	A	T	G
***02∶02*** 2	C	G	A	Y^3^	T	Y	G	G	S	C	C	R	I/D	M	C	T	I	C	R	A	I	A	G	T	R	C	K	A	G	S	A	T	R
***02∶05***	C	G	A	T	C	T	G	G	G	C	C	A	D	C	T	C	D	T	A	A	I	C	A	A	A	T	T	G	A	G	A	C	G
***03∶03***	C	G	A	T	T	T	G	G	C	C	C	A	D	C	C	T	I	C	G	A	I	A	G	T	G	C	T	A	G	G	A	T	G
***03∶04*** 2	C	G	A	T	T	T	G	G	S	C	C	A	D	C	C	T	I	C	G	A	I	A	G	T	G	C	T	A	G	G	A	T	G
***03∶16***	C	G	A	T	T	T	G	G	C	C	C	A	D	C	C	T	I	C	G	A	I	A	G	T	G	C	T	A	G	G	A	T	G
***04∶01***	C	G	A	T	T	T	G	G	G	C	C	A	D	C	C	T	I	C	G	A	I	A	G	T	A	C	T	A	G	G	A	T	G
***04∶08***	C	G	A	T	T	T	G	G	G	C	C	A	D	C	C	T	I	C	G	A	I	A	G	T	A	C	T	A	G	G	A	T	G
***05∶01***	T	G	A	T	T	T	G	G	G	C	C	A	D	C	T	C	D	T	A	A	I	C	A	A	A	T	T	G	A	G	A	T	G
***06∶02***	C	G	A	T	T	T	G	G	G	C	C	A	D	C	T	C	D	T	A	A	I	C	A	A	A	T	T	G	A	G	A	T	G
***06∶11***	C	G	A	T	T	T	G	G	G	C	C	A	D	C	T	C	D	T	A	A	I	C	A	A	A	T	T	G	A	G	A	T	G
***07∶01***	C	G	G	T	T	T	G	G	G	C	C	G	I	A	C	T	I	C	A	A	I	A	G	T	A	C	G	A	G	C	A	T	A
***07∶02***	C	G	A	C	T	T	G	G	G	C	C	G	I	A	C	T	I	C	A	A	I	A	G	T	A	C	G	A	G	C	A	T	A
***07∶04***	C	G	A	T	T	T	G	G	G	C	C	G	I	A	C	T	I	C	A	A	I	A	G	T	A	C	G	A	G	C	A	T	A
***07∶06***	C	G	A	T	T	C	G	G	G	C	C	G	I	A	C	T	I	C	A	A	I	A	G	T	A	C	G	A	G	C	A	T	A
***07∶18***	C	G	A	T	T	C	G	G	G	C	C	G	I	A	C	T	I	C	A	A	I	A	G	T	A	C	G	A	G	C	A	T	A
***08∶01***	T	G	A	T	T	T	G	G	G	C	C	A	D	C	T	C	D	T	A	A	I	C	A	A	A	T	T	G	A	G	A	T	G
***08∶02***	T	G	A	T	T	T	G	G	G	C	C	A	D	C	T	C	D	T	A	A	I	C	A	A	A	T	T	G	A	G	A	T	G
***08∶04***	T	G	A	T	T	T	G	G	G	C	C	A	D	C	T	C	D	T	A	A	I	C	A	A	A	T	T	G	A	G	A	T	G
***12∶02***	C	G	A	T	T	T	G	G	G	C	C	A	D	C	T	C	D	T	A	A	I	C	A	A	A	T	T	G	A	G	A	T	G
***12∶03***	C	G	A	T	T	T	G	G	G	C	C	A	D	C	T	C	D	T	A	A	I	C	A	A	A	T	T	G	A	G	A	T	G
***14∶02***	C	G	A	T	T	T	G	G	G	C	C	A	D	C	C	T	I	C	G	A	I	A	G	T	A	G	T	A	G	G	A	T	G
***15∶02***	C	G	A	T	T	T	G	G	G	C	C	A	D	C	T	C	D	T	A	A	I	C	A	A	A	T	T	G	A	G	A	T	G
***16∶01***	C	G	A	T	C	T	G	G	G	C	C	A	D	C	T	C	D	T	A	A	I	C	A	A	A	T	T	G	A	G	A	C	G
***16∶02***	C	G	A	T	C	T	G	G	G	C	C	A	D	C	T	C	D	T	A	A	I	C	A	A	A	T	T	G	A	G	A	C	G
***17∶01***	C	G	A	T	T	T	A	T	G	T	T	G	I	C	C	T	I	C	A	G	I	A	G	T	A	C	G	A	G	C	G	T	A

1 positions are given relative to the start of the HLA-C 3′ UTR.

2 *HLA-C* alleles C*02∶02 and C*03∶04 were found to have more than one 3′ UTR sequence.

3 where more than one allele has been observed alleles are reported using standard IUB ambiguity codes.

4 I refers to an insertion and D to a deletion.

### Estimation of LD Across the *HLA-C* 3′ UTR

In order to further investigate the relationships between the genetic variants identified within the *HLA-C* 3′ UTR, the ELB algorithm [17] was used to estimate the gametic phase of the genotyping data generated at all the polymorphic positions within this region. This allowed pairwise LD between each of these variants and the *HLA-C* alleles observed in each population group to be quantified, allowing the 3′ UTR sequences for each of the *HLA-C* alleles observed in both the Black and Caucasian population groups to be inferred. The *HLA-C* 3′ UTR sequences observed for each specific *HLA-C* allele are given in Table 1 (for the Black population group) and Table 2 (for the Caucasian population group). While the majority of alleles in both population groups were represented by a single 3′ UTR sequence, this was not always the case – with the Cw*02 alleles displaying multiple 3′ UTR sequences in both population groups. Similarly, the 3′ UTR sequence for a given allele was not necessarily the same in both population groups; with the Cw*02 alleles again providing the best example.

Given the observation that C*02∶10 could be associated with both an insertion and a deletion at position 263, we sought to establish the extent of LD between the *263* indel and the *HLA-C* alleles present in both population groups. These data are given in Table 3. Seventeen of the *HLA-C* alleles in the Black population group were in significant LD with the 263 indel. C*02∶02, C*03∶04, C*04∶01, C*07∶01, C*07∶02, C*07∶06, C*07∶18, C*17∶01 and C*18∶02 were in complete linkage with the insertion at position 263 (*263ins*); while C*05∶01, C*06∶02, C*08∶02, C*08∶04, C*12∶03, C*15∶05 and C*16∶01 were in complete linkage with the deletion at this position *(263del*). Although linkage was not complete, C*02∶10 was also found to be significantly associated with the *263del* allele. Fifteen of the *HLA-C* alleles in the Caucasian population group were found to be in significant LD with the 263 indel. C*02∶02, C*03∶03, C*03∶04, C*04∶01, C*07∶01 and C*07∶02 were in complete linkage with *263ins*, while C*05∶01, C*06∶02, C*08∶02, C*08∶04, C*12∶02, C*12∶03, C*15∶02, C*16∶01 and C*16∶02 were in complete linkage with *263del*.

**Table 3:** Linkage disequilibrium between the *263* indel and the *HLA-C* alleles present in the Black and Caucasian South African population groups.

	Black Individuals (n^1^ = 168)				Caucasian Individuals (n^1^ = 96)			
*HLA-C* Allele	3’ UTR Allele	Frequency^2^	D’^3^	p-value^4^	3’ UTR Allele	Frequency^2^	D’^3^	p-value^4^
01∶02	–	–	–	–	Ins	0.0208 (4^5^)	1.00	NS
02∶02	Ins	0.0089 (5)	1.00	<0.05	Ins	0.0625 (12)	1.00	<0.05
02∶05	Ins	0.0030 (1)	1.00	NS	Del	0.0052 (1)	1.00	NS
02∶10	Del	0.0327 (11)	0.33	<0.05	–	–	–	–
03∶02	Ins	0.0149 (5)	1.00	NS	–	–	–	–
03∶03	Ins	0.0030 (1)	1.00	NS	Ins	0.0625 (12)	1.00	<0.05
03∶04	Ins	0.0446 (15)	1.00	<0.01	Ins	0.0573 (11)	1.00	<0.05
03∶16	–	–	–	–	Ins	0.0052 (1)	1.00	NS
04∶01	Ins	0.1250 (42)	1.00	<0.01	Ins	0.0885 (17)	1.00	<0.01
04∶04	Ins	0.0030 (1)	1.00	NS	–	–	–	–
04∶08	–	–	–	–	Ins	0.0052 (1)	1.00	NS
05∶01	Del	0.0089 (3)	1.00	<0.05	Del	0.0521 (10)	1.00	<0.01
06∶02	Del	0.1488 (50)	1.00	<0.01	Del	0.0781 (15)	1.00	<0.01
06∶06	Ins	0.0030 (1)	1.00	NS	–	–	–	–
06∶11	Del	0.0030 (1)	1.00	NS	Del	0.0052 (1)	1.00	NS
07∶01	Ins	0.0714 (24)	1.00	<0.01	Ins	0.1771 (34)	1.00	<0.01
07∶02	Ins	0.0625 (21)	1.00	<0.01	Ins	0.1458 (28)	1.00	<0.01
07∶04	Ins	0.0149 (5)	1.00	NS	Ins	0.0104 (2)	1.00	NS
07∶06	Ins	0.0387 (13)	1.00	<0.01	Ins	0.0208 (4)	1.00	NS
07∶11	Del	0.0030 (1)	1.00	NS	–	–	–	–
07∶18	Ins	0.0417 (14)	1.00	<0.01	Ins	0.0156 (3)	1.00	NS
08∶01	Del	0.0030 (1)	1.00	NS	Del	0.0052 (1)	1.00	NS
08∶02	Del	0.0149 (5)	1.00	<0.01	Del	0.0208 (4)	1.00	<0.01
08∶04	Del	0.0298 (10)	1.00	<0.01	Del	0.0104 (2)	1.00	<0.05
12∶02	–	–	–	–	Del	0.0104 (2)	1.00	<0.05
12∶03	Del	0.0149 (5)	1.00	<0.01	Del	0.0208 (4)	1.00	<0.01
14∶02	Ins	0.0060 (2)	1.00	NS	Ins	0.0104 (2)	1.00	NS
15∶02	Del	0.0060 (2)	1.00	NS	Del	0.0260 (5)	1.00	<0.01
15∶05	Del	0.0089 (3)	1.00	<0.05	–	–	–	–
15:xx	Del	0.0060 (2)	1.00	NS	–	–	–	–
16∶01	Del	0.0714 (24)	1.00	<0.01	Del	0.0677 (13)	1.00	<0.01
16∶02	–	–	–	–	Del	0.0104 (2)	1.00	<0.05
17∶01	Ins	0.1131 (38)	1.00	<0.01	Ins	0.0052 (1)	1.00	NS
18∶01	Ins	0.0149 (5)	1.00	NS	–	–	–	–
18∶02	Ins	0.0298 (10)	1.00	<0.01	–	–	–	–

1 the total number of individuals genotyped in each population group.

2 the observed frequency of each two-locus haplotype.

3 Lewontin's D' measure of linkage disequilibrium [19].

4 p-values are calculated using an exact test for linkage disequilibrium [20], and are significant at p<0.05.

5 the number of chromosomes on which the two-locus haplotype was found to occur.

### Haplotype Structure within the *HLA-C* 3′ UTR

**Figure 1:**
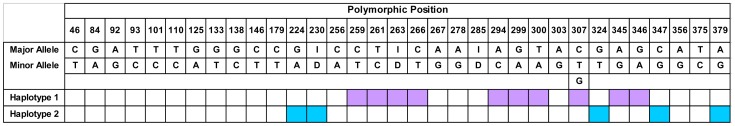
The haplotypes identified within the *HLA-C* 3′ UTR. The positions involved in the two haplotypes identified are indicated in colour. The haplotype encompassing the *263* indel is shown in pink, while the haplotype encompassing the *230* indel is shown in blue. The major and minor alleles at each position are also indicated. Positions were only included in the haplotypes if both D' and r^2^ measures of pairwise LD were equal to 1. Polymorphic positions are indicated by their position relative to the start of the *HLA-C* 3′ UTR.

LD analysis also identified the presence of two highly conserved, overlapping haplotypes involving multiple positions across the *HLA-C* 3′ UTR. The larger of these haplotypes involved ten positions (Figure 1; indicated in pink), spanning approximately 90 bases, and encompassing the indel at position 263. Another involved five positions (Figure 1; indicated in blue), spanning 155 bases of the *HLA-C* 3′ UTR, and encompassed the indel at position 230. Only positions with both D’ and r^2^ measures of pairwise LD equal to 1 were included within these haplotypes.

Because of complete linkage between the indels at positions 230 and 263, these haplotypes were found to be present in three possible combinations within all of the *HLA-C* alleles present in both population groups. These haplotypic combinations thus produced three “classes” of *HLA-C* 3′ UTR sequences, each defined by a specific combination of the two haplotypes observed. Class I corresponded to sequences with haplotypes defined by insertions at both *230* and *263*, class II to sequences with a deletion at *230* and an insertion at *263* and class III to sequences with deletions at both *230* and *263*.

### Linkage Disequilibrium between Specific *HLA-C* Alleles and the *-35* SNP

Genotyping of the *-35 *SNP (rs9264942) was also performed for 168 Black and 97 Caucasian South African individuals. The frequencies of the *35C* allele in the Black (0.348) and Caucasian (0.284) population groups were not significantly different (p = 0.13), and neither population group was found to deviate significantly from HWE at this position. This SNP has previously been shown to be in strong LD with a number of *HLA-C* alleles in individuals of Caucasian descent [3], [11], therefore the extent of LD between the *-35 *SNP and the *HLA-C* alleles present in both population groups was also evaluated (Table 4).

**Table 4:** Linkage disequilibrium between the *-35* SNP and the *HLA-C* alleles present in the Black and Caucasian South African population groups.

	Black Individuals (n^1^ = 168)				Caucasian Individuals (n^1^ = 97)			
*HLA-C* Allele	*-35* SNP Allele	Frequency^2^	D’^3^	p-value^4^	*-35* SNP Allele	Frequency^2^	D’^3^	p-value^4^
01∶02	–	–	–	–	C	0.0155 (3^5^)	0.65	<0.05
02∶02	C	0.0149 (5)	1.00	<0.05	C	0.0723 (14)	1.00	<0.01
02∶05	T	0.0030 (1)	1.00	NS	C	0.0052 (1)	1.00	NS
02∶10	T	0.0655 (22)	0.59	<0.05	–	–	–	–
03∶02	C	0.0149 (5)	1.00	<0.01	–	–	–	–
03∶03	C	0.0030 (1)	1.00	NS	T	0.0619 (12)	1.00	<0.05
03∶04	T	0.0446 (15)	1.00	<0.01	T	0.0567 (11)	1.00	<0.05
03∶16	–	–	–	–	T	0.0052 (1)	1.00	NS
04∶01	T	0.1250 (42)	1.00	<0.01	T	0.0876 (17)	1.00	<0.01
04∶04	T	0.0030 (1)	1.00	NS	–	–	–	–
04∶08	–	–	–	–	T	0.0052 (1)	1.00	NS
05∶01	C	0.0060 (2)	0.49	NS	T	0.0412 (8)	0.28	NS
06∶02	C	0.1488 (50)	1.00	<0.01	C	0.0773 (15)	1.00	<0.01
06∶06	C	0.0030 (1)	1.00	NS	–	–	–	–
06∶11	C	0.0030 (1)	1.00	NS	C	0.0052 (1)	1.00	NS
07∶01	T	0.0506 (17)	0.16	NS	T	0.1753 (34)	1.00	<0.01
07∶02	T	0.0595 (20)	0.86	<0.01	T	0.1443 (28)	1.00	<0.01
07∶04	T	0.0149 (5)	1.00	NS	T	0.0103 (2)	1.00	NS
07∶06	C	0.0387 (13)	1.00	<0.01	C	0.0206 (4)	1.00	<0.01
07∶11	T	0.0030 (1)	1.00	NS	–	–	–	–
07∶18	C	0.0417 (14)	0.89	<0.01	C	0.0155 (3)	1.00	<0.01
08∶01	T	0.0030 (1)	1.00	NS	T	0.0052 (1)	1.00	NS
08∶02	T	0.0119 (4)	0.42	NS	C	0.0103 (2)	0.31	NS
08∶04	C	0.0149 (5)	0.24	NS	C	0.0103 (2)	1.00	<0.05
12∶02	–	–	–	–	C	0.0103 (2)	1.00	<0.05
12∶03	C	0.0149 (5)	1.00	<0.01	C	0.0206 (4)	1.00	<0.01
14∶02	C	0.0060 (2)	1.00	<0.05	C	0.0103 (2)	1.00	<0.05
15∶02	T	0.0060 (2)	1.00	NS	T	0.0258 (5)	1.00	NS
15∶05	T	0.0089 (3)	1.00	NS	–	–	–	–
15:xx	T	0.0060 (2)	1.00	NS	–	–	–	–
16∶01	T	0.0655 (22)	0.76	<0.01	T	0.0670 (13)	1.00	<0.05
16∶02	–	–	–	–	T	0.0103 (2)	1.00	NS
17∶01	T	0.1131 (38)	1.00	<0.01	T	0.0052 (1)	1.00	NS
18∶01	T	0.0149 (5)	1.00	NS	–	–	–	–
18∶02	T	0.0298 (10)	1.00	<0.05	–	–	–	–

1 the total number of individuals genotyped in each population group.

2 the observed frequency of each two-locus haplotype.

3 Lewontin's D' measure of linkage disequilibrium [19].

4 p-values are calculated using an exact test for linkage disequilibrium [20], and are significant at p<0.05.

5 the number of chromosomes on which the two-locus haplotype was found to occur.

Of the 30 *HLA-C* alleles present in the Black population, 14 were found to be in significant LD with the *-35* SNP. C*02∶02, C*03∶02, C*06∶02, C*07∶06, C*07∶18, C*12∶03 and C*14∶02 were in complete linkage with the *-35C* allele, while C*03∶04, C*04∶01, C*17∶01 and C*18∶02 were in complete LD with the *-35T* allele (Table 4). While not complete, C*02∶10 (D’ = 0.59; p<0.05), C*07∶02 (D’ = 0.86; p<0.01) and C*16∶01 (D’ = 0.76; p<0.01) were also found to be in significant LD with the *-35T* allele. Of the 26 *HLA-C* alleles present in the Caucasian population, 15 were found to be in significant LD with the *-35* SNP. C*02∶02, C*06∶02, C*07∶06, C*07∶18, C*08∶04, C*12∶02, C*12∶03 and C*14∶02 were in complete linkage with the *-35C* allele, while C*03∶03, C*03∶04, C*04∶01, C*07∶01, C*07∶02 and C*16∶01 were in complete LD with the *-35T* allele (Table 4). Although not complete, C*01∶02 was also found to be in significant LD with the *-35C* allele (D' = 0.65, p<0.05).

### Linkage Disequilibrium between the *-35* SNP and the *263* Indel

Finally, to further investigate the relationship between the *-35* SNP and the *263* indel, LD between these polymorphisms was quantified in both the Black and Caucasian population groups. The *-35* SNP was only weakly associated with the *263* indel in both the Black (D' = 0.46; p<0.001) and the Caucasian (D' = 0.34; p<0.001) population groups. These polymorphisms have previously been shown to be in relatively strong LD (D’ = 0.75) in other Caucasian population groups [11]. In both population groups, the *-35C* allele was associated with *263 del* and the *-35T* allele with *263 ins*.

## Discussion

We examined genetic variation within the 3′ UTRs of the *HLA-C* alleles present in the Black and Caucasian South African population groups and investigated the relationship between these variants and a SNP 35 kb upstream of the *HLA-C* locus. The data confirmed the presence of the *263* indel (rs67384697) in both population groups, as well as that of other polymorphisms previously identified within this region in other populations [11], [22], [23]. The persistence of strong LD was observed between particular 3′ UTR polymorphisms in both population groups and the underlying haplotypic structure of the region was described for each *HLA-C* allele. Finally, these data demonstrated that the *-35* SNP (rs9624942) is not in strong LD with the *263* indel in either the Black or the Caucasian South African population; and as such, is not an appropriate marker for this indel in either of these groups. These findings have important implications for our understanding of the regulation of HLA-C expression and the resulting impact on HIV-1 disease progression.

Previous investigations of genetic variability within the *HLA-C* 3′ UTR in other populations have identified numerous polymorphisms other than the *263* indel in this region [11], [22], [23]. Consistent with their findings, our analysis of the same region identified 33 of these polymorphisms in the Black South African population. The variation identified within the Caucasian South African population represented only a subset of that seen in the Black population group. However, the additional polymorphisms observed in the Black population group were not unique to this population, having been previously described in other studies [11], [22], [23]. A number of these polymorphisms were found to deviate from HWE in both population groups - although different polymorphisms were seen to deviate from HWE between the Black and Caucasian population groups. While deviation from HWE can often be attributed to genotyping errors, this is unlikely to be the case in this instance, as all genotyping was performed by direct sequencing. In light of the finding that a subset of these polymorphisms are unique to specific *HLA-C* alleles and given the differences observed in the *HLA-C* allele distributions between the two population groups, these deviations from HWE most likely reflect the influence of random genetic drift.

When we examined the variants present within the 3′ UTR sequences from Caucasian individuals in terms of their association with specific *HLA-C* alleles, we again observed a number of similarities with previous descriptions of variability within the region [11], [22], [23]. Each *HLA-C* allele was found to be in complete linkage with a specific *263* allele, although eleven of these associations did not reach statistical significance. Given the high number of *HLA-C* alleles observed in this population, this lack of statistical significance was most likely a consequence of the low frequencies of these alleles within the population. Similarly, all but one of the *HLA-C* alleles observed in the Black population were in complete LD with a specific *263* allele. Several of these associations also did not reach statistical significance, again most likely as a result of the low frequencies of these alleles within the population.

As previously observed [11], [22], [23], the majority *HLA-C* alleles observed within the Caucasian population group were represented by a single, distinctive 3′ UTR sequence. However, contrary to prior observations, several different sequences were observed for the C*02∶02 allele in this population group – none of which corresponded to sequences previously reported for this allele. Nonetheless, despite this variability, none of the 3′ UTR sequences observed for C*02∶02 differed with respect to their *263* indel allele. A similar pattern was observed for the 3′ UTR sequences of the *HLA-C* alleles present in the Black population group – with C*02∶02 again showing a high degree of variability within this population group, but without differing with respect to the *263* indel allele observed. However, one allele present only in the Black population group (HLA C*02∶10), was found to be associated with both the *263ins* and *263del* alleles. Coupled with the finding that the 3′ UTR sequence observed for C*02∶05 differed between the two population groups, these data may suggest that a diversity of genetic variability exists within the Cw 02 alleles that cannot be adequately described using the current commonly used *HLA-C* SBT genotyping methods.

LD analysis between all the polymorphisms present in the *HLA-C* 3′ UTR revealed the *263* indel to be at the centre of a large haplotype involving the same ten polymorphic positions in both the Black and Caucasian populations. This indel is thought to regulate differential *HLA-C* expression by disrupting a putative miRNA binding site [11]. It has previously been shown to be in complete linkage with the SNPs at positions 256, 261 and 266; and as none of the SNPs produced any significant change in luciferase activity when analysed independently, the indel is thought to be responsible for any differences seen in HLA-C expression [11]. However, all analyses of alterations in luciferase activity involving the indel included concomitant modifications at all four positions, and all four polymorphisms occur within the putative miR-148a/miR-148b binding site [11], allowing for the possibility that concomitant changes at these positions may act synergistically to disrupt miRNA binding.

Similarly, the SNP at position 307 was also previously shown to be potentially disruptive of a putative miRNA binding site, but when analysed independently did not produce any significant changes in luciferase activity [11]. However, if analysed in haplotypic combination with the SNPs at positions 299, 300 and 303, which occur within the same putative mi-657 binding site [11]; a significant difference in expression may potentially be observed, as the presence of multiple mismatched bases within the same binding site would most likely increase the probability of disruption of miRNA binding relative to modification at a single position. Additionally, the observation that these variants always occur in combination with each other, and could thus collectively potentially prevent binding of more than one miRNA, would further suggest that the presence of the haplotype (rather than any one single polymorphism) is responsible for the differential expression of *HLA-C* alleles observed. However, further functional studies would be required to test this hypothesis.

A second large haplotype encompassing the indel at position 230 and four other SNPs was also observed in both populations. The presence of an insertion at position 230, coupled with a guanine at 224, introduces at additional putative miR-181a binding site into the *HLA-C* 3′ UTR [11]. As a result, when both indels are present in a population, the two haplotypes overlap to produce three separate “classes” of *HLA-C* 3′ UTR sequences that are all potentially subject to varying degrees of miRNA-mediated gene regulation. *HLA-C* 3′ UTR sequences with deletions at both positions 230 and 263 would therefore potentially disrupt the binding of four miRNAs (miR-181a, miR-148a/miR-148b, miR-657 and miR-181a* [11]), while those sequences with insertions would be inhibited at the same positions. Sequences with a deletion at position 230 and insertion at 263 would disrupt two putative miRNA binding sites (miR-148a/miR-148b and miR-657 [11]), and thus potentially exhibit a phenotype of intermediate expression. These three separate lineages could thus account for the variances previously observed in HLA-C expression [3], [11], [24]. Again, however, further functional studies are necessary to test this hypothesis.

The suggestion that the overlap of the two haplotypes described could potentially account for observed variances in HLA-C expression is consistent with the previous findings of Corrah *et al*. [24], who prior to the identification of the *263* indel, used monoclonal antibodies to examine HLA-C expression in relation to *-35* SNP genotype [24]. They attributed the association between the *-35T* allele and higher HIV-1 viral set point observed in Caucasian individuals[1], [3]–[7] to the especially low expression of HLA-Cw*07 alleles, which are particularly common in individuals of European descent [13]. This association is not seen in African-American populations[7]–[9], where HLA-Cw*07 is less prevalent. All the HLA-Cw*07 sequences analysed during the course of our investigation had insertions at both indel positions, and would thus (under the aforementioned model) be subject to especially strong miRNA-mediated inhibition. This could also partially account for variances in the distribution of HLA-C expression levels observed in other functional studies [10], [11].

This association between the *-35* SNP and HLA-C expression [3], was previously thought to be responsible for the differences in HIV-1 viral set point observed in Caucasian individuals. However, the *263* indel is now considered to be the actual variant responsible for the differential regulation of HLA-C expression, which, in turn, is thought to be responsible for the variation seen in HIV-1 viral set point [11]. The association observed between the *-35* SNP and in HIV-1 viral set point has since been attributed to strong LD between the SNP and indel [11].

Examination of the extent of LD between the *-35* SNP and *263* indel in both the Black and Caucasian populations revealed LD between them (and all the other SNPs in the haplotype) to be weak in both groups. However when linkage between the *-35* SNP and specific *HLA-C* alleles was examined, LD was found to be complete and significant for fourteen alleles in the Caucasian population. This was consistent with previous reports in other Caucasian populations [3], [11]. LD was not found to be significant for more than 50% of the *HLA-C* alleles in the Black population group. This is consistent with observations in African-American populations, where common *HLA-C* alleles show no significant LD with the *-35* SNP; and for alleles that do show significant LD, linkage is not complete [10].

Thus while the *-35* SNP may be indicative of specific *HLA-C* alleles in the Caucasian population, it cannot be regarded as an effective marker for the *263* indel in either the Black or Caucasian South African population groups. The *-35* SNP was also not in strong LD with the *230* indel in either population group (data not shown). Thus, as has been shown in the African-American population [10], this SNP is unlikely to associate with either HLA-C expression or HIV-1 viral set point in these groups. However, this is yet to be confirmed. Whether the *-35* SNP associates with any other as yet unidentified polymorphisms that influence HIV-1 disease progression by alternative mechanisms may warrant further investigation.

In conclusion, these data provide the first description of variation in the regulatory regions of *HLA-C* for the Black and Caucasian South African populations. Furthermore, the data from the Black population are the first for a sub-Saharan African population. These data allow for a description of the haplotypic patterns within the *HLA-C* 3′ UTR and identify two overlapping haplotypes within this region - which we hypothesize may act independently and synergistically to influence miRNA regulation of HLA-C expression. Concomitantly, we demonstrate that the *-35* SNP is not in strong LD with either haplotype (in either population) and as such is unlikely to be an appropriate marker for HLA-C expression in either of these populations. Even in the absence of supporting expression data, these findings provide important insights into genetic variability within the regulatory regions of *HLA-C*, that have potential implications for our understanding of the regulation of HLA-C expression and its impact on HIV-1 disease progression in the populations occupying the region worst affected by this epidemic.

## Supporting Information

Table S1 **The *HLA-C* alleles present in both the Black and Caucasian population groups and their frequencies.** The observed frequency of each allele was calculated according to the formula n/2n, where n refers to the number of times each allele was observed within the given population group and 2n refers to the total number of chromosomes considered within each population group. The allelic frequencies are representative of 168 Black individuals and 97 Caucasian individuals.
(PDF)

Table S2 **The polymorphic positions within the *HLA-C* 3′ UTR and their minor allele frequencies in the Black and Caucasian population groups.** All positions are given relative to the start of the *HLA-C* 3′ UTR. The allelic frequencies are representative of 168 Black individuals and 96 Caucasian individuals. The p-values given are for a two-sided Fisher’s exact test and only significant values (p<0.05) shown.
(PDF)
