# Supplementary material for: Genetic Variability in Markers of HLA-C Expression in Two Diverse South African Populations
Source: PLoS One. 2013 Jul 5;8(7):e67780. doi: 10.1371/journal.pone.0067780 (PMC3702582; doi:10.1371/journal.pone.0067780)
Supplement: Table S1 — The HLA-C alleles present in both the Black and Caucasian population groups and their frequencies. The observed frequency of each allele was calculated according to the formula n/2n, where n refers to the number of times each allele was observed within the given population group and 2n refers to the total number of chromosomes considered within each population group. The allelic frequencies are representative of 168 Black individuals and 97 Caucasian individuals. (PDF) [file pone.0067780.s001.pdf]

Table S1: *HLA-C* allele frequencies in the Black and Caucasian population groups

***HLA-C* Allele**	**Black Individuals**		**Caucasian Individuals**	
	**n**	**Frequency**	**n**	**Frequency**
01:02	0	-	4	0.021
02:02	5	0.015	14	0.072
02:05	1	0.003	1	0.005
02:10	26	0.077	0	-
03:02	5	0.015	0	-
03:03	1	0.003	12	0.062
03:04	15	0.045	11	0.057
03:16	0	-	1	0.005
04:01	42	0.125	17	0.088
04:04	1	0.003	0	-
04:08	0	-	1	0.005
05:01	3	0.009	10	0.052
06:02	50	0.149	15	0.077
06:06	1	0.003	0	-
06:11	1	0.003	1	0.005
07:01	24	0.071	34	0.175
07:02	21	0.063	28	0.144
07:04	5	0.015	2	0.010
07:06	13	0.039	4	0.021
07:11	1	0.003	0	-
07:18	14	0.042	3	0.016
08:01	1	0.003	1	0.005
08:02	5	0.015	4	0.021
08:04	10	0.030	2	0.010
12:02	0	-	2	0.010
12:03	5	0.015	4	0.021
14:02	2	0.006	2	0.010
15:02	2	0.006	5	0.026
15:05	3	0.009	0	-
15:xx	2	0.006	0	-
16:01	24	0.071	13	0.067
16:02	0	-	2	0.010
17:01	38	0.113	1	0.005
18:01	5	0.015	0	-
18:02	10	0.030	0	-
