# Supplementary material for: Genetic Variability in Markers of HLA-C Expression in Two Diverse South African Populations
Source: PLoS One. 2013 Jul 5;8(7):e67780. doi: 10.1371/journal.pone.0067780 (PMC3702582; doi:10.1371/journal.pone.0067780)
Supplement: Table S2 — The polymorphic positions within the HLA-C 3′ UTR and their minor allele frequencies in the Black and Caucasian population groups. All positions are given relative to the start of the HLA-C 3′ UTR. The allelic frequencies are representative of 168 Black individuals and 96 Caucasian individuals. The p-values given are for a two-sided Fisher’s exact test and only significant values (p<0.05) shown. (PDF) [file pone.0067780.s002.pdf]

**Table S2:** Polymorphic positions within the *HLA-C* 3' UTR

| Position | dbSNP ID | Alleles | Minor Allele Frequency | | p-value |
|---|---|---|---|---|---|
| | | | Black Individuals | Caucasian Individuals | |
| 46 | rs1049853 | C/T | 0.067 | 0.089 | - |
| 84 | rs1049724 | A/G | 0.003 | 0.000 | - |
| 92 | rs1049709 | A/G | 0.052 | 0.177 | <0.001 |
| 93 | rs1065711 | C/T | 0.061 | 0.151 | 0.001 |
| 101 | rs3176007 | C/T | 0.046 | 0.083 | - |
| 110 | rs41289069 | C/T | 0.135 | 0.047 | 0.001 |
| 125 | rs1049668 | A/G | 0.117 | 0.005 | <0.001 |
| 133 | rs1049663 | G/T | 0.117 | 0.005 | <0.001 |
| 138 | rs1049650 | C/G | 0.065 | 0.094 | - |
| 146 | rs116229144 | C/T | 0.117 | 0.005 | <0.001 |
| 179 | rs1049579 | C/T | 0.117 | 0.005 | <0.001 |
| 224 | rs1094 | A/G | 0.379 | 0.401 | - |
| 230 | rs35877659 | G/- | 0.379 | 0.401 | - |
| 256 | rs1130592 | A/C | 0.256 | 0.373 | - |
| 259 | rs3207555 | C/T | 0.307 | 0.260 | - |
| 261 | rs3207561 | C/T | 0.307 | 0.260 | - |
| 263 | rs67384697 | G/- | 0.354 | 0.313 | - |
| 266 | rs1130586 | C/T | 0.351 | 0.313 | - |
| 267 | rs1130580 | A/G | 0.253 | 0.286 | - |
| 278 | rs1130576 | A/G | 0.122 | 0.005 | <0.001 |
| 285 | rs60637457 | ACTT/- | 0.003 | 0.000 | - |
| 294 | rs1130559 | A/C | 0.351 | 0.313 | - |
| 299 | rs1130558 | A/G | 0.351 | 0.313 | - |
| 300 | rs1130554 | A/T | 0.351 | 0.313 | - |
| 303 | rs1130552 | A/G | 0.065 | 0.135 | 0.006 |
| 307 | rs1071643 | C/G/T | 0.003 | 0.010 | - |
| 324 | rs1130538 | G/T | 0.393 | 0.401 | - |
| 345 | rs116302614 | A/G | 0.351 | 0.313 | - |
| 346 | rs115906458 | A/G | 0.351 | 0.313 | - |
| 347 | rs3189472 | C/G | 0.393 | 0.401 | - |
| 356 | rs115510686 | A/G | 0.122 | 0.005 | <0.001 |
| 375 | rs114027487 | C/T | 0.074 | 0.078 | - |
| 379 | rs1049281 | A/G | 0.393 | 0.401 | - |
